# The Effectiveness of Post-Vaccination and Post-Infection Protection in the Hospital Staff of Three Prague Hospitals: A Cohort Study of 8-Month Follow-Up from the Start of the COVID-19 Vaccination Campaign (COVANESS)

**DOI:** 10.3390/vaccines10010009

**Published:** 2021-12-22

**Authors:** Marek Petráš, Ivana Králová Lesná, Livia Večeřová, Elka Nyčová, Jana Malinová, Petr Klézl, Martina Nezvedová, Rachel Elizabeth White, Roman Máčalík, Jana Dáňová, Alexander M. Čelko, Věra Adámková

**Affiliations:** 1 Third Faculty of Medicine, Charles University, 100 00 Prague, Czech Republic; macalik@ulz.cz (R.M.); jana.danova@lf3.cuni.cz (J.D.); martin.celko@lf3.cuni.cz (A.M.Č.); 2 Institute for Clinical and Experimental Medicine, 140 21 Prague, Czech Republic; ivka@ikem.cz (I.K.L.); vead@ikem.cz (V.A.); 3 First Faculty of Medicine, Charles University and Military University Hospital, 121 08 Prague, Czech Republic; 4 Bulovka University Hospital, 180 00 Prague, Czech Republic; livia.vecerova@bulovka.cz (L.V.); elka.nycova@bulovka.cz (E.N.); 5 Královské Vinohrady University Hospital, 100 34 Prague, Czech Republic; jana.malinova@fnkv.cz (J.M.); petr.klezl@fnkv.cz (P.K.); martina.nezvedova@fnkv.cz (M.N.); rachel.white@fnkv.cz (R.E.W.)

**Keywords:** effectiveness, COVID-19 vaccination, hospital workers, reinfection, breakthrough infection

## Abstract

Continuous assessment of the effectiveness of approved COVID-19 vaccines is crucial to gain an insight into the longer-term impact on health outcomes, and eventually boosting public confidence. For this reason, we conducted a multicenter, retrospective cohort study using data on infection and vaccination rates among employees of three Prague hospitals in the period between 27 December 2020 and 31 August 2021. The post-vaccination and post-infection protectiveness were assessed in a total of 11,443 hospital workers who were followed up for more than 14 days either after their Comirnaty vaccination or study enrolment, depending on their previous SARS-CoV-2 infection. The effectiveness of full vaccination against any SARS-CoV-2 infection achieved 88.3% (83.2–91.8%) over the eight months of follow-up, a figure not much different from the 92.5% (76.5–97.6%) level of protection built by a previous infection. Despite this, the post-vaccination level of protection declined to about 65% between June and August. No case of breakthrough infection was registered among hospital workers having received one or two vaccine doses more than three months after previous infection. The eight-month effectiveness of the Comirnaty vaccine exhibited a declining trend requiring a new booster dose. The need for vaccination in the previously infected employees was not demonstrated conclusively in this study.

## 1. Introduction

On 21 December 2020, the European Medicines Agency (EMA) issued an Emergency Use Authorization (EUA) for the Comirnaty vaccine (Pfizer-BioNTech) as the first vaccine against COVID-19 indicated for use in those over 16 years of age. Subsequently, the same approval was granted to other vaccines, Spikevax (Moderna), Vaxzevria (Oxford/AstraZeneca), and the Janssen COVID-19 vaccine. The promising efficacy of these vaccines established in short-term clinical trials provided a good starting point for overcoming the pandemic caused by severe acute respiratory syndrome coronavirus 2 (SARS-CoV-2).

Similar to other countries, both health care workers and the elderly, as those being at high risk of contracting COVID-19, were prioritized for the start of the vaccination campaign in the Czech Republic. The initial real-word experience of immunization was consistent with the results of clinical trials. However, a reduced level of protection of the complete, typically 2-dose vaccination schedule was documented within the next few months by observational studies performed in different countries [1,2,3,4]. The reduction was ascribed not only to replacement of the SARS-CoV-2 variant but, also, to the likely declining effectiveness of any EMA-approved vaccine.

In an effort to assess the effectiveness of COVID-19 vaccination in the staff of three Prague hospitals over the first eight months from the start of the vaccination campaign in the Czech Republic, we conducted a retrospective cohort study based on hospital databases. We investigated the vaccine effectiveness achieved in both those previously SARS-CoV-2 infected, and uninfected hospital workers (HWs). We sought to compare the levels of protection afforded by naturally acquired and vaccine-induced immunity to find a difference, if any.

## 2. Materials and Methods

### 2.1. Study Population

The COVANESS study (COVID Vaccine effective NESS) was conducted in the hospital staff, predominantly health-care workers, of three Prague hospitals: Bulovka University Hospital (BUH), Královské Vinohrady University Hospital (KVUH), and the Institute for Clinical and Experimental Medicine (IKEM). Between 1 March 2020 and 21 August 2021, a total of 12,249 full- or part-time employees were enrolled in this retrospective cohort study. Using their registers, the participating hospitals created an electronic database of the personal data of their employees, their COVID-19 vaccination records, and medical records, including laboratory-confirmed disease with the results of on-site real-time polymerase chain reaction (RT-PCR) tests.

The source data to be analyzed contained the employees’ identification codes, sex, age in the year 2020, and their employment status (full-time or part-time; health-care worker or another profession). The PCR tests were performed according to the hospital screening policy, based on the risk of SARS-CoV-2 exposure in employees. All RT-PCR tested employees had their results, including the recorded testing date. When SARS-CoV-2 infection was confirmed, the record contained the cycle threshold (Ct) value, the presence/absence of at least one symptom of COVID-19, including possible required hospitalization. For each positive test, a single Ct was calculated as the arithmetic mean across the detected genes, then the lowest value was calculated across positives in the respective infection episode to reflect the highest viral load within an episode. As identification of the particular SARS-CoV-2 variant was missing in most laboratory records, this information was omitted. The database was supplemented with the person’s vaccination status, i.e., date(s) of vaccine administration and dose, including the vaccine name.

### 2.2. Study Design

The start of COVANESS was defined by the first day of the vaccination against COVID-19 (27 December 2020), and the follow-up was terminated on 31 August 2021. A pre-study period enabled us to identify the employees with previous SARS-CoV-2 infection who could be vaccinated later. To determine the vaccine effectiveness against breakthrough infections, participants with no previous SARS-CoV-2 infection were arranged into three cohorts according to their vaccination status (unvaccinated, partially, and fully vaccinated). The previously infected employees were used to establish the effect of post-infection protection against re-infection with no subsequent vaccination or with partial or full vaccination.

The study was approved by the Ethics Committee of the Third Faculty of Medicine, Charles University in Prague. The final report of COVANESS followed the Strengthening the Reporting of Observational Studies in Epidemiology guidelines (Supplementary file S1). The study sponsors had no role in study design, data collection, data analysis, data interpretation, or the writing of the article.

The primary objective of this study was to evaluate the effectiveness of both post-vaccination and post-infection protection against laboratory-confirmed SARS-CoV-2 infection irrespective of COVID-19 symptoms in employees with >14 days of follow-up from enrolment or vaccination. The terms “post-vaccination” and “post-infection” protection referred to periods of more than 14 days after vaccine administration and 90 days after previous SARS-CoV-2 infection, respectively [5]. The secondary objectives focused on vaccine effectiveness in specific populations grouped by sex, stratified age, profession, and hospital.

### 2.3. Statistical Analysis

To evaluate the primary and secondary endpoints, Poisson regression was used. The log-rank test was applied to assess the equality of failure functions. The crude incidence rate ratio (IRR) was calculated from the incidence rates of SARS-CoV-2 infection in the vaccinated or naturally immune versus the incidence rates in the unvaccinated/infection-naïve participants. The IRR was also mutually adjusted for the covariates of sex, stratified age (≤30 years, 30–45 years, 45–60 years, and >60 years), health-care workers and other professions, full- and part-time, and by hospital. The post-vaccination and post-infection levels of protection were estimated from crude and adjusted IRRs as follows: 100% × (1-IRR).

A sensitivity analysis was performed for those employees who had had at least one RT-PCR test within the study period. The impact of symptomatic SARS-CoV-2 infection on vaccination effectiveness was conducted with PCR-positive employees having the disease and exhibiting at least one COVID-19 symptom.

The persistence of post-vaccination protection was estimated in the months of February and March, and in the periods of April-May and June-August. The analyses were conducted in a group of those having PCR tests in the above periods, while not previously infected and being either more than 14 days after the second vaccine dose or unvaccinated with >14-day follow-up. The effectiveness of full vaccination was estimated from the odds ratio (OR) adjusted for the same covariates as those used in the primary objective and calculated using logistic regression. All tests were two-tailed, and the level of significance was set at 0.05. Statistical analyses and regressions were performed using Prism 9 (GraphPad Software, Inc., San Diego, CA, USA) and STATA/SE version 17 software (StatCorp, Lakeway Drive, TX, USA), respectively.

## 3. Results

Between 27 December 2020 and 31 August 2021, a total of 11,443 HWs were enrolled in the COVANESS study and followed up for an average of 220 days, with a standard deviation (SD) of 60 days. The employees were mostly women (74.3%), and their mean age including SD was 41.5 ± 13.8 years. They were age-stratified as follows: ≤30 years, >30–45 years, >45–60 years, and >60 years. At least one RT-PCR test was performed in 2750 employees (24%) over the study period, with a RT-PCR positive result obtained in 549.

**Figure 1:**
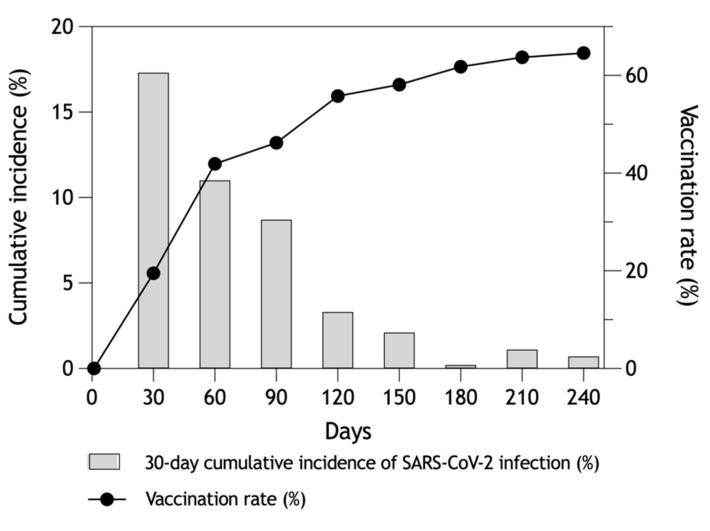
Vaccination rate and 30-day cumulative incidence of SARS-CoV-2 infection in HWs.

The cumulative incidence rate of SARS-CoV-2 infection at 30-day intervals showed a decreasing trend (Figure 1). This was associated with an increasing cumulative vaccination rate of employees immunized with the Comirnaty vaccine. The post-vaccination level of protection was assessed in those receiving the Comirnaty vaccine because only 0.3% of the employees received another available vaccine (Vaxzevria, SpikeVax or Janssen).

During the study period, 65.6% of HWs were immunized with at least one dose of the Comirnaty vaccine and 64.8% with two doses. The vaccination rate was higher in men (66.7%) than in women (64.2%). Full vaccination was achieved in 58.7% of those below 45 years of age, while both doses of the Comirnaty vaccine were administered to 73.8% of the older employees, and even as many as 77.8% of HWs aged >60 years. A significantly higher rate of 66.4% was found in health-care workers as compared with other workers (60.2%). Moreover, 71.6% of HWs with full-time employment contract received both vaccine doses. The vaccination coverage differed among the three participating hospitals, being highest at IKEM (74.6%), followed by KVUH (62.6%) and BUH (61.5%).

SARS-CoV-2 infection was confirmed by the Ct-value in 537 results out of a total of 549 findings (97.8%), and in three of four cases of reinfections. The infection was diagnosed in 371 unvaccinated, 123 partly vaccinated, and 55 fully vaccinated HWs irrespective of the interval post-vaccination or follow-up. Compared with the unvaccinated HWs (Ct = 24.7), the median Ct value of 26.8 was significantly higher any time following the second dose of Comirnaty, but breakthrough infections observed >14 days after vaccination were documented by a lower median Ct of 25.0. Moreover, no post-vaccination time-dependence of Ct values was proved. The total of three reinfections documented by the Ct value (median 24.8) were insufficient to assess the difference between primary infection and reinfection.

Of the total of 549 infections, symptomatic disease was registered in 292 (53.2%) and asymptomatic infections in 44 employees (8.0%). The records of any symptoms of SARS-CoV-2 infection in the remaining 213 cases were unavailable. Hospitalization was reported in only 18 employees, of which number 17 had not been vaccinated, and 13 days after a single-dose vaccination in one case.

**Figure 2:**
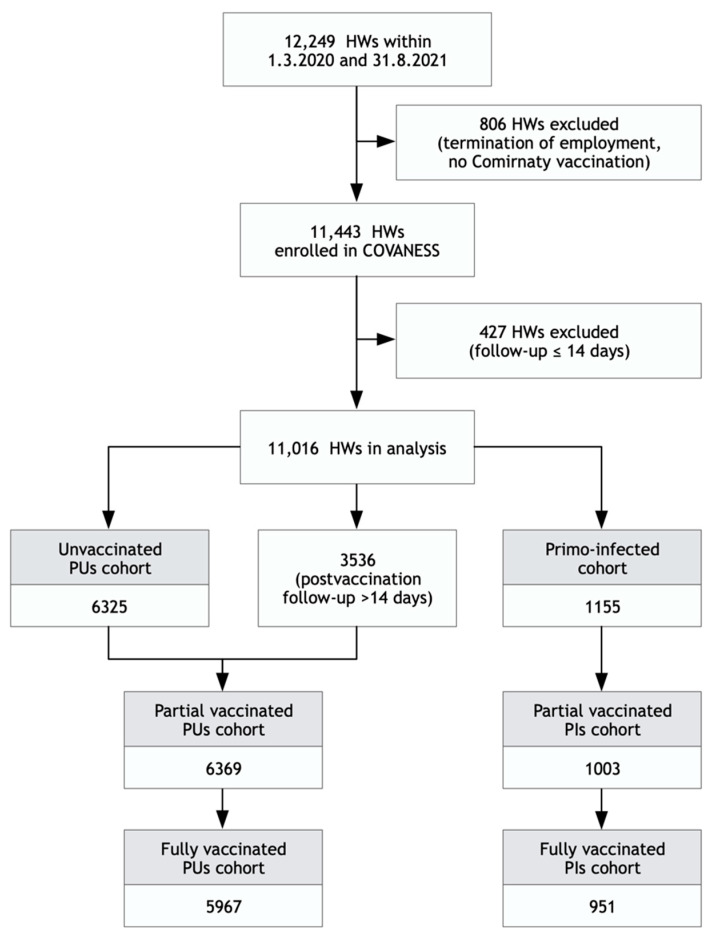
Study flowchart: HWs: hospital workers; PUs: previously uninfected; PIs: previously infected.

The analysis of vaccine effectiveness was conducted in 11,016 HWs followed up for more than 14 days irrespective of their vaccination status, with a total of 254 laboratory-confirmed infections. The arrangement of HWs into cohorts was based on the presence/absence of a previous SARS-CoV-2 infection (Figure 2).

The size and attack rates of recorded SARS-CoV-2 infections, breakthrough infections or reinfections in all HWs grouped by sex, stratified age groups, health care, and type of employment contract, including their mean age and follow-up duration, are summarized for each study cohort (Table 1). The mean interval between the first and second doses in the fully vaccinated cohort was 24.4 ± 10 days in previously uninfected (PU) participants, and 27.6 ± 8.3 days in those previously infected (PI) with SARS-CoV-2. Follow-up was significantly shorter in the partly vaccinated HWs of both cohorts due to the recommended interval of the double dose vaccination schedule. 

Partial vaccination of PU employees showed short-term effectiveness of 47.7% (19.2–66.2%), regardless of the infection-related symptoms (Table 2). However, the rate increased to 75.4% (0.7–93.9%) in PIs, with only two breakthrough infections reported within 15–30 days after single-dose administration at an interval of <3 months since any previous infection. No SARS-CoV-2 infection was diagnosed in participants vaccinated longer than three months after their previous infection. The estimated effectiveness against symptomatic SARS-CoV-2 infections increased to 76.4% (46.0–89.7) and 100% in the partly vaccinated PU and PI subjects, respectively.

Vaccine effectiveness against any SARS-CoV-2 infection achieved 88.3% (83.2–91.8%) in the fully vaccinated HWs, irrespective of their RT-PCR test results within the eight months of study follow-up. Analysis of sensitivity in the fully vaccinated participants showed an effectiveness of 89.7% (85.3–92.9) in those having at least one RT-PCR test, and 91.7% (85.7–95.2%) in those diagnosed with symptomatic COVID-19.

Among the fully vaccinated HWs with previous SARS-CoV-2 infection, there was only one case of breakthrough infection diagnosed 11 days after the second dose of the Comirnaty vaccine. Therefore, the effectiveness against any SARS-CoV-2 infection determined at 14 days post-immunization was estimated at 100% (99.9−100%) in the fully vaccinated participants over an average follow-up of 154 days.

**Figure 3:**
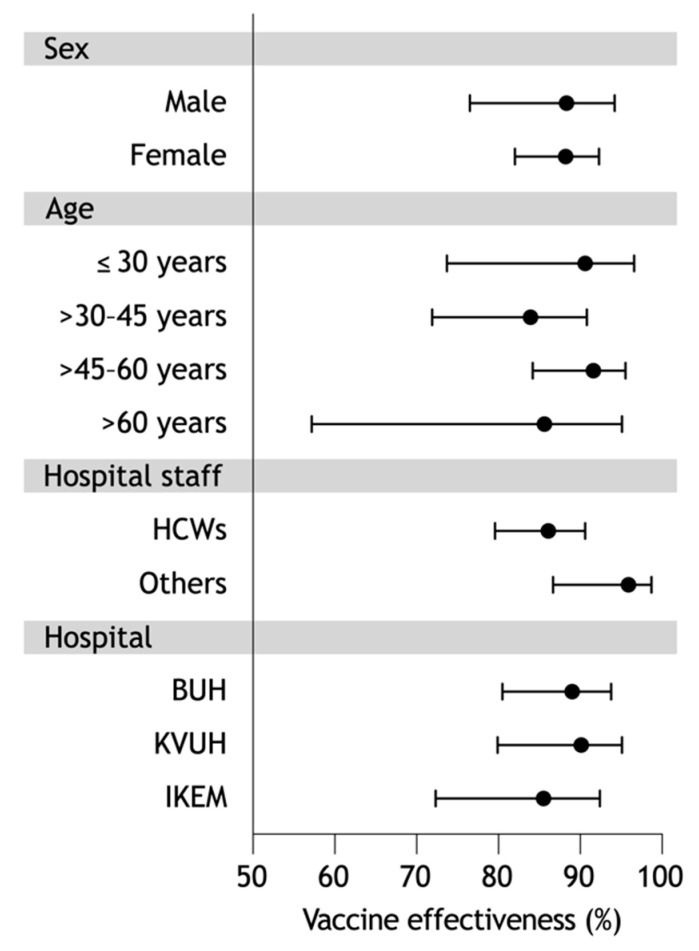
Vaccine effectiveness in specific PU group within 8-month follow-up: HCWs: health-care workers; BUH: Bulovka University Hospital; KVUH: Královské Vinohrady University Hospital; IKEM: Institute for Clinical and Experimental Medicine.

The effectiveness against any SARS-CoV-2 infection did not differ between men and women who were not previously infected (Figure 3). A decrease to 85.6% (57.2–95.1%) was seen in those over 60 years of age. The significantly low attack rate of SARS-CoV-2 infection observed in other employees contributed to the higher vaccine effectiveness of 95.9% (86.7–98.7%), compared with that of 86.1% (79.6–90.6%) in health-care workers. Despite of the highest vaccination rate in HWs at IKEM, the 85.5% effectiveness of full vaccination was the lowest among the participating hospitals.

**Figure 4:**
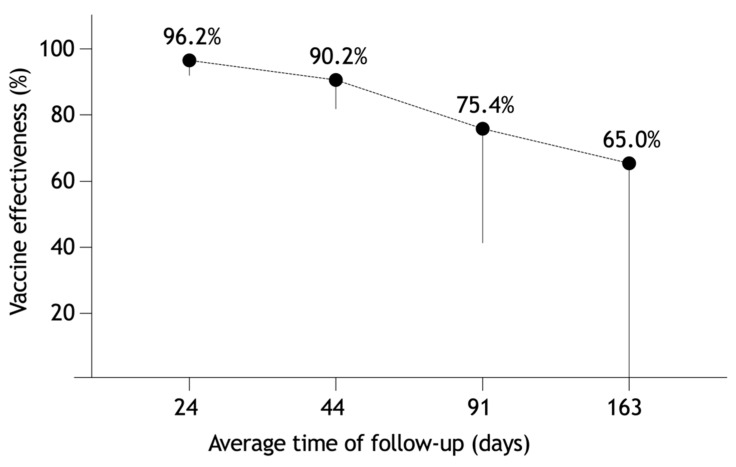
Persistence of vaccine effectiveness.

The effectiveness decreased with follow-up duration (Figure 4). While the first months confirmed a stable protective effect of 96.2% (91.6–98.7%) in February and 90.2% (81.5–95.7%) in March, the effect decreased in the subsequent months to 75.4% (40.8–94.2%) between April and May, and to 65% (<0 to 96.6%) between June and August. These figures were consistent with the more rapid decline in the infection rates in unvaccinated participants (from 60.0% to 1.9%) than in the fully vaccinated ones (from 6.8% to 1.0%).

**Figure 5:**
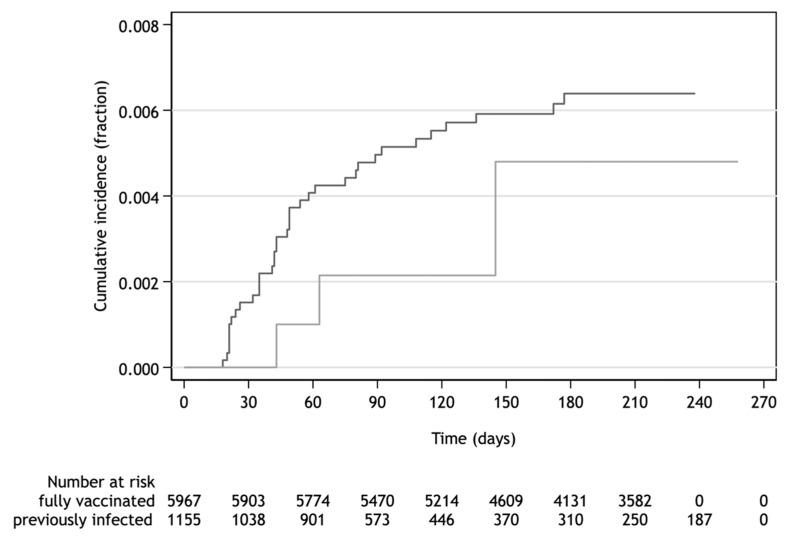
Cumulative incidence of any SARS-CoV-2 infection among fully vaccinated and previously infected.

The HWs with naturally acquired protection after previous SARS-CoV-2 infection confirmed by RT-PCR were at low risk of re-infection. The level of protection by post-infection immunity achieved 92.5% (76.5−97.6%) regardless of COVID-19 symptoms. The interval between primary infection and re-infection was in the range of 103–176 days. The adjusted incidence rate ratio of SARS-CoV-2 infection of the unvaccinated previously infected to fully vaccinated participants was 0.67 (0.20–2.17). This ratio demonstrated no difference of in the protective effect acquired either by infection or vaccination as documented by cumulative incidence over up to 258 days of follow-up (Figure 5).

## 4. Discussion

A decreasing incidence of SARS-CoV-2 infection was observed in accordance with the increasing vaccination rate of hospital staff during the first eight months after beginning of available vaccination against COVID-19. Only 0.3% of HWs were vaccinated with another available vaccine against COVID-19 than Comirnaty vaccine. Therefore, the study effectiveness of 88.3% was measured by fully immunization with Comirnaty vaccine administered to 65% of hospital staff. This outcome was consistent with early and later results of published studies conducted after the initiation of worldwide vaccination against COVID-19 [6,7,8,9,10].

The occurrence of any SARS-CoV-2 infection more than 14 days after partial immunization was reduced by only 48%, a figure lower than that reported in other studies [2,11]. This discrepancy could be explained by the short-term, 26-day, follow-up, since a longer follow-up duration has been shown to be associated with an increasing effect of the single dose [10,12]. Moreover, partial and full vaccination with the Comirnaty vaccine reduced the risk of symptomatic COVID-19 by 76% and 92%, respectively.

However, the effectiveness against any SARS-CoV-2 infection declined to 75% and 65% at 3 and 5.4 months after complete immunization with the Comirnaty vaccine. A similar trend of waning post-vaccination protection by about 40% has been reported by other authors [1,2,3,4]. This effect is most often ascribed to the change in SARS-CoV-2 variants, especially the Delta variant (B.1.617.2) [13]. As in other countries, variant replacement has been observed in the Czech Republic [14].

Whether the replacement is the main reason of the waning level of protection is not currently clear due to the observation of a significant decrease in virus-neutralizing antibodies within approximately six months after full vaccination [15]. Therefore, one may reasonably assume that variant replacement as well as waning post-vaccination immunity may contribute to the higher rates of breakthrough infection in the fully vaccinated.

The declining level of post-vaccination protection was also suggested by the Ct values of breakthrough SARS-CoV-2 infections. While all 54 cases of breakthrough infection were documented by a median Ct value of 26.8 irrespective of the time of vaccination, 35 of them diagnosed later than 14 days after complete vaccination dropped median Ct to 25.0 in conformity with that seen in the unvaccinated HWs.

A high, 92.5%, level of protection against any SARS-CoV-2 reinfection among the previously infected HWs was determined during the 8-month follow-up. This result was consistent with the pooled 87% efficacy of naturally acquired immunity reported in 15 studies [16]. Furthermore, no marked difference was found between vaccine effectiveness and post-infection level of protection up to 258 days of follow-up. This finding was in partial contradiction to the outcomes of more recent studies demonstrating either higher levels of protection by post-infection immunity than the effectiveness of full vaccination [17], or quite the reverse [18]. Unfortunately, the body of our current knowledge is not large enough to recognize a higher risk of reinfection or breakthrough infections in previously infected or fully vaccinated immune individuals, especially in the long run.

Even if the contribution of vaccination to naturally immune individuals is not yet clear, approximately 75% of the previously infected HWs decided to undergo complete vaccination. A single dose conferred them a higher level of protection compared with those previously uninfected (75% vs. 48%) during follow-up with a median of 27 days. Only three cases of infection among a total of 1003 HWs were reported at any time point after the single vaccine dose administered earlier than three months after the primary infection. Otherwise, no case of breakthrough infection was found if the single dose was given later than three months after primary infection. It can be assumed that this early vaccination (<3 months after SARS-CoV-2 infection) could not meet the requirement of booster vaccination, whereby the next dose is administered at least three or more months after building primary immunity.

Unfortunately, the impact of the second vaccine dose was not evident in COVANESS, although no case of SARS-CoV-2 infection was identified among the previously infected participants. The short-term follow-up of single-dose immunization did not enable us to evaluate any possible difference between the one- and two-dose vaccination schemes.

One may reasonably assume that infection is able to induce an adequate immune response similar to that elicited by primary vaccination. Similar to common vaccination, subsequent single-dose vaccination is likely to reinforce current immunity as a booster if administered in at an appropriate interval, i.e., longer than three months. The improvement in protection through single-dose vaccination in the previously infected was demonstrated by recent outcome studies [19,20]. The need for a second vaccine dose is questionable, since the outcomes of serological studies suggested no contribution to humoral or cellular immunity [21,22].

Our study has some limitations. The analyses were primarily conducted with all eligible HWs regardless of the outcome of their RT-PCR tests on the assumption that HWs would always have a PCR test at their respective hospital, if necessary. Although it cannot be excluded that an employee could be infected and go undetected by PCR testing, our sensitivity analysis confirmed the consistency of test-dependent and test-independent results. Therefore, if a bias exists at all, then it should be presumably low.

Regardless of the post-vaccination or post-infection immunity, a higher risk of SARS-CoV-2 infection was present in health care and full-time workers. Conversely, a slightly lower incidence was found in KVUH employees. Still, any potential risk of bias was damped by mutual adjustment.

There was only one case of hospitalization for COVID-19 among hospital staff after the administration of the first vaccine dose. Therefore, it was not possible to evaluate the effectiveness or protectiveness against hospitalization. The HWs’ comorbidities were not registered, hence no specific analyzes were performed to assess the impact of vaccination on study participants with comorbidities.

The impact of SARS-CoV-2 variants replacement on vaccination effectiveness was not investigated since the swab samples were only sporadically sequenced for variant identification. Therefore, the particular variants predominance was only estimated from other sources available in the Czech Republic [14,23].

## 5. Conclusions

The rapid vaccination rate of hospital staff significantly reduced the risk of community spread of SARS-CoV-2 infection among both immunized and unimmunized employees. Nevertheless, a waning post-vaccination level of protection should be expected, as shown by COVANESS, with an approximately 30% decrease in the level of protection observed later than four months after full immunization with the Comirnaty vaccine.

The naturally acquired protection after a previous SARS-CoV-2 infection showed sufficient effectiveness equivalent to that of 2-dose vaccination. However, a need for vaccination with one or two doses in the previously infected subjects was not supported by this study. Single- dose vaccination at an interval of >3 months after a previous SARS-CoV-2 infection should be considered if necessary.

## Figures and Tables

**Table 1:** Baseline characteristics of study participants in cohorts.

Characteristics		Unvaccinated (PUs ^1^)	Partially Vaccinated (PUs)	Fully Vaccinated (PUs)	Previously Infected	Partially Vaccinated (PIs ^2^)	Fully Vaccinated (PIs)
**Mean age ± SD ^3^ (years)**		39.4 ± 13.9	43.7 ± 13.5	43.8 ± 13.4	42.2 ± 13.3	42.4 ± 13.4	42.6 ± 13.2
**Mean follow-up ± SD (days)**		135.9 ± 90.8	25.9 ± 18.0	185.1 ± 51.5	119.5 ± 77.0	28.1 ± 12.9	154.0 ± 51.9
**All employees (% PCR+ ^4^)**		6325 (3%)	6369 (0.4%)	5967 (0.6%)	1155 (0.3%)	1003 (0.2%)	951 (0%)
**Sex** **(% PCR+)**	**Female**	4777 (3.1%)	4671 (0.4%)	4353 (0.6%)	893 (0.1%)	755 (0.1%)	715 (0.0%)
	**Male**	1548 (2.8%)	1698 (0.3%)	1614 (0.6%)	262 (0.8%)	248.(0.4%)	236 (0.0%)
**Age ** **(% PCR+)**	**≤30 years**	1920 (2.4%)	1264 (0.2%)	1158 (0.3%)	265 (0.4%)	236 (0.4%)	218 (0.0%)
	**>30–45 years**	2232 (3.3%)	2148 (0.4%)	2022 (0.7%)	401 (0.0%)	338 (0.0%)	320 (0.0%)
	**>45–60 years**	1641 (3.8%)	2141 (0.5%)	2046 (0.6%)	389 (0.5%)	324 (0.3%)	313 (0.0%)
	**>60 years**	532 (2.1%)	816 (0.4%)	741 (0.7%)	100 (0.0%)	105 (0.0%)	100 (0.0%)
**Employees ** **(% PCR+)**	**HCWs ^5^**	4391 (3.2%)	4792 (0.4%)	4493 (0.7%)	950 (0.3%)	846 (0.1%)	803 (0.0%)
	**Others**	1934 (2.7%)	1577 (0.3%)	1474 (0.2%)	205 (0.0%)	157 (0.6%)	148 (0.0%)
**Employment status (% PCR+)**	**Full-time**	4561 (3.9%)	5549 (0.4%)	5223 (0.7%)	1063 (0.2%)	945 (0.2%)	899 (0.0%)
	**Part-time**	1764 (0.9%)	820 (0.1%)	744 (0.3%)	92 (1.1%)	58 (0.0%)	52 (0.0%)
**Hospital ** **(% PCR+)**	**BUH ^6^**	2441 (3.5%)	2283 (0.4%)	2175 (0.6%)	430 (0.0%)	375 (0.0%)	353 (0.0%)
	**KVUH ^7^**	2741 (2.5%)	2564 (0.5%)	2350 (0.4%)	461 (0.4%)	367 (0.0%)	352 (0.0%)
	**IKEM ^8^**	1143 (3.3%)	1522 (0.1%)	1442 (0.9%)	264 (0.4%)	261 (0.8%)	246 (0.0%)

^1^ PUs: previously uninfected; ^2^ PIs: previously infected; ^3^ SD: standard deviation; ^4^ PCR+: positive result of RT-PCR; ^5^ HCWs: health care workers; ^6^ BUH: Bulovka University Hospital; ^7^ KVUH: Královské Vinohrady University Hospital; ^8^ IKEM: Institute for Clinical and Experimental Medicine.

**Table 2:** Incidence rate ratios and vaccine effectiveness in any SARS-CoV-2 infection (participants with more than 14 days of follow-up).

Immunization Status		PCR+ ^1^	IR ^2^ per 100.000 Person-Days	cIRR ^3^ (95% CI^4^)	cVE ^5^ (95% CI)	aIRR ^6^ (95% CI)	aVE ^7^ (95% CI)
**Unvaccinated**		192	22.33	ref ^8^		ref	
**Previously infected**		3	2.22	0.10 (0.02–0.29)	90.3 (71.1–98.0)	0.08 (0.02–0.24)	92.5 (76.5–97.6)
**Partially vaccinated**	**PU ^9^**	23	14.07	0.63 (0.39–0.97)	37.0 (2.7–61.0)	0.52 (0.34–0.81)	47.7 (19.2–66.2)
	PI* ^10^	2	7.11	0.32 (0.04–1.17)	68.2 (−16.5–96.2)	0.25 (0.06–0.99)	75.4 (0.7–93.9)
**Fully vaccinated**	**PU**	36	3.25	0.15 (0.10–0.21)	85.4 (79.1–90.1)	0.12 (0.08–0.17)	88.3 (83.2–91.8)
	**PI**	0	0.00	0.00 (0.00–0.11)	100 (88.6–100)	0.00 (0.00–0.00)	100.0 (99.9–100)

^1^ PCR+: positive result of RT-PCR; ^2^ IR: incidence rate; ^3^ CI: confidence interval; ^4^ cIRR: crude incidence rate ratio; ^5^ cVE: crude vaccine effectiveness; ^6^ aIRR: adjusted incidence rate ratio; ^7^ aVE: adjusted vaccine effectiveness; ^8^ ref: reference group; ^9^ PUs: previously uninfected; ^10^ PIs: previously infected; * vaccinated any time after SARS-CoV-2 infection; Note: Mutually adjusted for sex, stratified age, health-care worker vs. another profession, full- vs. part-time status, and hospital.

## Data Availability

Data available on request due to restrictions privacy. The data presented in this study are available on request from the corresponding author. The data are not publicly available due to confidential agreement with participating hospitals.

## Supplementary Materials

The following are available online at https://www.mdpi.com/article/10.3390/vaccines10010009/s1, Supplementary file S1: STROBE Statement—Checklist of items that should be included in reports of cohort studies.
